# Characterization of a New Flavone and Tyrosinase Inhibition Constituents from the Twigs of *Morus alba* L.

**DOI:** 10.3390/molecules21091130

**Published:** 2016-09-02

**Authors:** Long Zhang, Guanjun Tao, Jie Chen, Zong-Ping Zheng

**Affiliations:** 1 State Key Laboratory of Food Science and Technology, Jiangnan University, Wuxi 214122, Jiangsu, China; longzhang0109@gmail.com (L.Z.); tgjfirst@hotmail.com (G.T.); chenjie@jiangnan.edu.cn (J.C.); 2 Synergetic Innovation Center of Food Safety and Nutrition, Jiangnan University, Wuxi 214122, Jiangsu, China

**Keywords:** *Morus alba*, twigs, phenolic compounds, tyrosinase inhibition

## Abstract

The twigs of *Morus alba* L. were found to show strong tyrosinase inhibition activity, and the responsible active components in the extract were further investigated in this study. A flavone, named morusone (**1**), and sixteen known compounds **2**–**17** were isolated from *M. alba* twigs and their structures were identified by interpretation of the corresponding ESI-MS and NMR spectral data. In the tyrosinase inhibitory test, the compounds steppogenin (IC_50_ 0.98 ± 0.01 µM), 2,4,2′,4′-tetrahydroxychalcone (IC_50_ 0.07 ± 0.02 µM), morachalcone A (IC_50_ 0.08 ± 0.02 µM), oxyresveratrol (IC_50_ 0.10 ± 0.01 µM), and moracin M (8.00 ± 0.22 µM) exhibited significant tyrosinase inhibition activities, much stronger than that of the positive control kojic acid. These results suggest that *M. alba* twig extract should served as a good source of natural tyrosinase inhibitors for use in foods as antibrowning agents or in cosmetics as skin-whitening agents.

## 1. Introduction

In agricultural and food products, enzymatic browning caused by tyrosinase (EC 1.14.18.1) in fruits and vegetables usually impairs the color attributes and sensory properties of these products and leads to a significant decrease in their quality, and eventually results in a dramatic reduction in nutritional and market values [1]. On the other hand, tyrosinase is responsible for the synthesis of melanin in animals and human beings [2,3]. Melanin can protect the human skin from ultraviolet damage, but excessive melanin pigmentation may cause serious esthetic problems [4]. As enzymatic browning in foods and hyperpigmentation of human skin are mostly undesirable, application of tyrosinase inhibitors to control undesirable browning in food industry and to provide skin whitening or to suppress excessive melanin production in the cosmetic and pharmaceutical industry has attracted great attention. Although a number of tyrosinase inhibitors from natural sources have been discovered in recent years [5,6], source limitations still one of the major obstacles in the exploitation of natural tyrosinase inhibitors. Therefore, there is still a great demand from the food and cosmetic industry to identify more abundant and safe tyrosinase inhibitors of natural origin. Mulberry tree (*Morus alba* L.) is one of *Morus* species which belongs to the Moraceae family. It is widely distributed in Asia and is cultivated in China, Korea and Japan for different purposes. Various parts of this plant including the roots, fruits, twigs, leaves, and root barks have been used as traditional Chinese medicine for centuries. The root bark of *M. alba* (called “Sang-Bai-Pi” in China) has been used as a medicinal herb for humans to treat fever, improve eyesight, protect the liver, strengthen joints, lower blood pressure, and facilitate the discharge of urine [7]. The leaves of *M. alba* have been used for a long time in traditional medicine for the treatment of fever, protection of the liver and lowering blood pressure [8]. *M. alba* twigs have been widely used for the treatment of aching and numbness of joints in oriental medicine and are known to have potential health benefits in folk medicine against diabetes, stroke, cough, and beriberi [9]. Most studies on the phytochemistry and bioactivity for *M. alba* have mainly focused on the leaves, root barks, and fruits, whereas few studies have paid attention to the constituents and biological activities for the twigs, which are usually discarded as agricultural waste. Recently, some phenolic compounds, such as oxyresveratrol 3′-*O*-β-d-glucoside, oxyresveratrol, resveratrol, moracin M, maclurin, rutin, isoquercitrin, and morin, were isolated from mulberry twigs [9,10], and morin was reported to be a natural antioxidant and tyrosinase inhibitor [10]. Mulberry young twig extract has exhibited potent inhibitory effects on mushroom, murine, and human tyrosinase and melanin synthesis in B-16 melanoma cells [11]. On the other hand, mulberry twig extract and oxyresveratrol from its extract have been reported to show remarkable antibrowning effects on cloudy apple juices and fresh-cut apples slices in combination with ascorbic acid [12]. Therefore, *M. alba* twigs and its constituents could be a promising natural source for development as dietary supplements, antibrowning agents, and cosmetic whitening products.

Although some phenolic compounds have been isolated from the twigs of *M. alba* and its extract had been reported to have tyrosinase inhibition and antibrowning effects, to our knowledge, except for oxyresveratrol and mulberroside A the principal constituents of *M. alba* twigs responsible for the tyrosinase inhibition have not yet been clearly identified until now. Thus, the aim of this study was to investigate in detail the components responsible for the inhibitory activities on tyrosinase.

## 2. Results and Discussion

**Figure 1:**
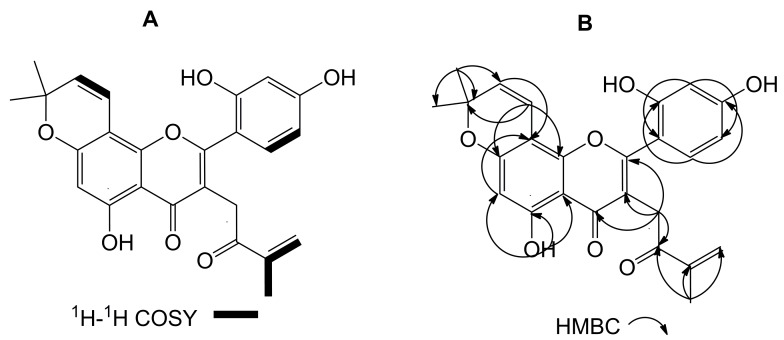
^1^H-^1^H COSY (**A**) and key HMBC (**B**) correlations of the new compound **1**.

**Figure 2:**
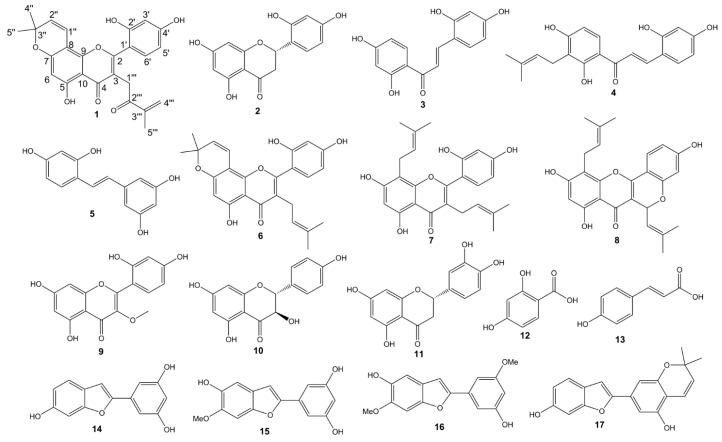
Structures of compounds from the twigs of *M. alba*.

Compound **1** was obtained as a pale yellow amorphous powder. Its negative-ion HRESI-MS gave a molecular ion peak at *m/z* 433.1270 ([M − H]^−^), which suggested its molecular formula to be C_25_H_22_O_7_. The UV spectrum of compound **1** showed a maximum absorption at 270 nm. The IR spectrum of this compound displayed absorption bands at 3385 cm^−1^ (OH), 1655 cm^−1^ (C=O), 1617 and 1575 cm^−1^ (C=C). Its ^1^H-NMR and ^13^C-NMR spectra suggested that compound **1** was likely a flavone. Its ^1^H-NMR spectrum clearly exhibited the presence of three hydroxyl signals (δ_H_ 12.926 (1H, s, OH) and 8.874 ppm (2H, s, 2 × OH)), four aromatic proton signals (δ_H_ 7.217, 6.642, 6.544, and 6.486 ppm), four olefin proton signals (δ_H_ 6.173, 6.088, 5.812, and 5.678 ppm), one methylene signal (δ_H_ 3.851 ppm) and three methyl signals [δ_H_ 1.814 (CH_3_ × 1) and 1.456 ppm (CH_3_ × 2)]. The singlet at δ_H_ 6.173 ppm (1H, s) was assignable to the proton at C-6 position of a 1,2,4,5,6-pentasubstituted flavone A ring. Three aromatic signals at δ_H_ 7.217 (1H, d, *J* = 8.4 Hz), 6.544 (1H, d, *J* = 2.0 Hz) and 6.486 ppm (1H, dd, *J* = 8.4, 2.0 Hz) formed one ABX system and were assignable to the protons at C-6′, 3′ and 5′ position of a 2′,4′-dihydroxy-substituted flavone B ring, respectively. The proton signals at δ_H_ 6.642 (1H, d, *J* = 10.0 Hz), 5.678 (1H, d, *J* = 10.0 Hz), and 1.456 (6H, s) suggest the possible presence of one 2,2-dimethylpyrano unit, which was formed by cyclization of an isoprenyl unit with the flavonoid skeleton. The proton signals at δ_H_ 6.088 (1H, s), 5.812 (1H, d, *J* = 1.4 Hz), and 1.814 (3H, s), together with a carbonyl group at *δ*_C_ 198.36 formed an isopropenyl methyl ketone group. In the ^1^H-^1^H COSY spectrum (Figure 1A), the signal at δ_H_ 1.814 (3H, s) showed the correlations with signals at δ_H_ 6.088 (1H, s) and 5.812 (1H, d), which indicated the fragment of CH_3_−C=CH_2_, the signal at δ_H_ 6.642 (1H, d) exhibited the correlations with signals at δ_H_ 5.678 (1H, d), suggesting the presence of the fragment of –CH=CH–, and the signal at δ_H_ 7.217 (1H, d) had the correlations with signals at δ_H_ 6.486 (1H, dd) suggesting one 1,2,4-dihydroxy-substituted aromatic ring in the structure. Its ^13^C-NMR spectrum revealed the presence of 25 carbons, including 12 aromatic carbons, six olefinic carbons, two carbonyl carbons, one oxygenated alkyl carbon, one methylene, and three methyl groups. Furthermore, in its HMBC spectrum (Figure 1B), the proton at δ_H_ 6.642 (H-1′′) exhibited correlations with the carbons at δ_C_ 1602.9 (C-7), 153.39 (C-9), and 78.97 (C-3′′), which allowed the assignment of the 2,2-dimethylpyrano unit to the position C-7 and C-8 of the A ring (Figure 2). On the other hand, proton at δ_H_ 3.851 (H-1′′′) exhibited correlations with the carbons at δ_C_ 163.42 (C-2), 117.77 (C-3), 182.79 (C-4), and 198.36 (C-2′′′), suggesting that the isopropenyl methyl ketone group was attached to the position C-3 of the C ring. Moreover, the proton at δ_H_ 1.814 (H-5′′′) showed correlations with the carbons at δ_C_ 198.36 (C-2′′′), 145.13 (C-3′′′), and 125.03 (C-4′′′), also confirming that the modified isoprenyl unit was linked to the C-3 position of the C ring. The hydroxyl proton at δ_H_ 12.926 (OH-5) showed correlations with the carbons at δ_C_ 162.69 (C-5), 100.04 (C-6), and 105.28 (C-10) and the proton at δ_H_ 6.172 (H-6) exhibited the corrections with carbons at δ_C_ 101.93 (C-8), 160.29 (C-7), 162.69 (C-5), and 105.28 (C-10). The proton at δ_H_ 6.544 (H-3′) had corrections with carbons at δ_C_ 132.41 (C-6′) and 108.53 (C-5′), the proton at δ_H_ 7.217 also exhibited correlations with carbons at δ_C_ 157.56 (C, C-2′) and 161.97 (C-4′), the proton at δ_H_ 6.486 (H-5′) showed corrections with carbons at δ_C_ 112.23 (C-1′) and 104.18 (C-3′), and the proton at δ_H_ 6.544 (H-3′) showed corrections with carbons at δ_C_ 112.23 (C-1′), 108.53 (C-5′), 161.97 (C-4′), and 157.56 (C-2′), confirming the presence of a 1′,2′,4′-dihydroxy-substituted B ring. Accordingly, compound **1** was identified as 2-(2,4-dihydroxyphenyl)-5-hydroxy-8,8-dimethyl-3-(3-methyl-2-oxo-but-3-enyl)-8*H*-pyrano[2,3-*f*]chromen-4-one, and named morusone. The structure and all assignments of compound **1** were further determined by detailed HSQC and HMBC analysis.

The known compounds from the twigs of *M. alba* were identified by comparing their ESI-MS, ^1^H-NMR and ^13^C-NMR data with the literature as steppogenin (**2**) [13], 2,4,2′,4′-tetrahydroxy-chalcone (**3**) [14], morachalcone A (**4**) [14], oxyresveratrol (**5**) [13], morusin (**6**) [15], kuwanon C (**7**) [16], cyclomulberrin (**8**) [17], 5,7,2′,4′-tetrahydroxy-3-methoxyflavone (**9**) [18], dihydrokaempferol (**10**) [19], eriodictyol (**11**) [20], 2,4-dihydroxybenzoic acid (**12**) [21], *p*-coumaric acid (**13**) [22], moracin M (**14**) [14], moracin J (**15**) [23], moracin B (**16**) [24], and moracin D (**17**) [25] (Figure 2). These seventeen compounds included five flavones, four benzofuran derivatives, three flavanones, two chalcones, two phenolic acids, and one stilbene derivate. Among the sixteen known compounds, 5,7,2′,4′-tetrahydroxy-3-methoxyflavone (**9**) and eriodictyol (**11**) were found for the first time in *M. alba* species, whereas steppogenin (**2**), 2,4,2′,4′-tetrahydroxychalcone (**3**), morachalcone A (**4**), kuwanon C (**7**), cyclomulberrin (**8**), dihydrokaempferol (**10**), 2,4-dihydroxybenzoic acid (**12**), *p*-coumaric acid (**13**), moracin J (**15**), moracin B (**16**), and moracin D (**17**) were firstly found from *M. alba* twigs. 2,4,2′,4′-Tetrahydroxychalcone and morachalcone A, two best powerful tyrosinase inhibitors from natural sources, had been indentified in the root bark [26] and 2,4,2′,4′-tetrahydroxychalcone had been found in leaves in this plant before [27]. In this study, both of them were simultaneously identified in the twigs.

The tyrosinase inhibitory activity of compounds **1**–**17** was compared using a tyrosinase inhibition assay and their activities were expressed as IC_50_ values. Among these seventeen compounds, steppogenin, 2,4,2′,4′-tetrahydroxychalcone, morachalcone A, oxyresveratrol, and moracin M showed much stronger mushroom tyrosinase inhibitory activities than kojic acid (Table 1). Although the extract of *M. alba* twigs and some of its components (such as oxyresveratrol and mulberroside A) had been reported to have anti-melanogenic effects, the principal constituents (except for oxyresveratrol and mulberroside A) of *M. alba* twigs responsible for the tyrosinase inhibition have not yet been clearly identified. This study simultaneously identified five compounds with high tyrosinase inhibitory activity in the twigs of *M. alba*. These results suggested that *M. alba* twig extract should be served as a good source of natural tyrosinase inhibitors for use in foods as antibrowning agents or in cosmetics as skin-whitening agents. On the other hand, the new compound, morusone (**1**) showed weak tyrosinase inhibitory activity (Table 1). Although it did not inhibit mushroom tyrosinase effectively, compared to other compounds, a flavonoid attached by an isopropenyl methyl ketone group is relatively rare, and could provide a reference for structural identification of other compounds in the future. This study has been the first to simultaneously identify 2,4,2′,4′-tetrahydroxychalcone and morachalcone A in *M. alba* twigs, although both of them had been found from the roots of *M. nigra* by our group before [14]. A structure-activity relationship study for flavonoids and their glucosides, stilbenes and their glucosides, 2-arylbenzofuran derivatives, coumarin glycosides had been discussed in detail in our previous studies [13,14,28], but several aspects for the structure-activity relationship study need to be emphasized and expanded as follows: (1) an unsubstituted resorcinol group at the 2′- and 4′-OH in the B-ring of flavonoids was very important for their tyrosinase inhibitory activities, and changes in both the number and location of hydroxyl substituents caused changes in the tyrosinase inhibitory activities. Examples were compounds **2** and **11**, the former was 2′- and 4′-OH substituted, whereas the latter was 3′- and 4′-OH substituted. Although they only differ in their hydroxyl group substitution pattern, they demonstrated tremendously different tyrosinase inhibition activities, with the former being more than 100-fold stronger than the latter; (2) the substitution of hydroxyl, methoxyl, isoprenyl, and glucose groups at the C-3 position of the C-ring usually significantly decreased the tyrosinase inhibition activity. For example, substitution of the methoxyl group at the C-3 position of the C ring led to much weaker tyrosinase inhibitory activity for compound **9** (compared with norartocapein [28]); (3) the presence of isoprenyl groups on the A or B-ring usually decreased the tyrosinase inhibition activity, which was affected by the positions and the forms of isoprenyl groups, such as cyclization with hydroxyl groups of flavonoids and substitution by hydroxyl groups or changes into isopropenyl methyl ketone; (4) 2-arylbenzofuran derivatives usually showed moderate tyrosinase inhibitory activity, and the factors affecting this tyrosinase inhibitory activities were similar to those of the flavonoid derivatives. For example, the cyclization with a hydroxyl group decreases the tyrosinase inhibitory activity (compound **17**); (5) many single resorcinol derivatives usually exhibited weak tyrosinase inhibitory activities, although some of them have 2 and 4-OH substitutions. Examples were compounds **12**, 2,4-dihydroxybenzaldehyde and 2,4-dihydroxyacetophenone [29], all of which showed weak tyrosinase inhibitory activities with IC_50_ values over 200 µM.

## 3. Materials and Methods

### 3.1. General Information

Mushroom tyrosinase (5771 units/mg), l-tyrosine, methanol-*d*_6_, acetone-*d*_6_ and kojic acid were purchased from Sigma Chemical Co (St. Louis, MO, USA). Dichloromethane (CH_2_Cl_2_), dimethyl sulfoxide (DMSO), 95% ethanol (EtOH), methanol (MeOH), sodium dihydrogen orthophosphate (NaH_2_PO_4_·2H_2_O), formic acid, and anhydrous di-sodium hydrogen phosphate (Na_2_HPO_4_) were purchased from Sinopharm Chemical Reagent Co., Ltd. (Suzhou, China). HPLC grade solvents were purchased from J&K Scientific (Newark, DE, USA). Silica gel (200–300 mesh) for column chromatography and TLC plates (HSGF254) were purchased from Yantai Jiangyou Silicone Development Co., Ltd. (Yantai, China). Sephadex LH-20 was purchased from GE Healthcare Bio-Sciences AB (Uppsala, Sweden). D101 macropore adsorptive resin was purchased from Anhui Sanxing Resin Technology Co., Ltd. (Anhui, China). Analytical HPLC was carried out on a Waters 1525 system (Waters, Milford, MA, USA) equipped with a 2487 dual-wavelength detector and the Empower Pro software. Alltima C_18_ column (250 mm × 4.6 mm, 5 μm, Delta Technical Products Co., Des Plaines, IL, USA) was used for analytical HPLC. ^1^H-NMR, ^13^C-NMR, HSQC and HMBC data were acquired on a 400 DRX NMR spectrometer (Bruker, Colorado Springs, CO, USA). Molecular weights of compounds were analyzed on Waters Maldi Syapt Q-Tof mass spectrometer. Spectrophotometric measurements for the tyrosinase inhibition assay were taken on a UV-5300PC spectrophotometer (Metash Instrument Co., Ltd., Shanghai, China).

### 3.2. Plant Material

The twigs of *M. alba* were purchased from Bozhou Chinese Herbal Pieces Co. Ltd. (Bozhou, Anhui, China). Voucher specimen (accession number 20160401) was deposited at State Key Laboratory of Food Science and Technology, Jiangnan University (Wuxi, Jiangsu, China).

### 3.3. Extraction and Isolation

*M. alba* twig powder (10.0 kg) was extracted by 70% ethanol (20.0 L × 3). The extraction solution was filtered and all the filtrates were combined and concentrated under vacuum using a rotatory evaporator at 50 °C to remove ethanol. The extract (217.2 g) was subjected to silica gel (200–300 mesh) column chromatography and eluted with dichloromethane (CH_2_Cl_2_), CH_2_Cl_2_/MeOH (50:1, 25:1, 10:1, 5:1, *v/v*), which led to five fractions (Fr.1–5). Fr.2 (CH_2_Cl_2_/MeOH, 10:1) was further subjected to D101 macropore adsorptive resin column chromatography by successively eluted with different proportions of water/ethanol mixtures (H_2_O/EtOH, *v/v*, 7:3, 3:2, 1:19) to yield four fractions (SubFrs.1–4). SubFr.1 (H_2_O/EtOH, 7:3, 1.6 g) was separated by a Sephadex LH-20 column and eluted by MeOH/H_2_O (1:1) to give two fractions (Fr.A1 and A2). Fr.A1 (0.5 g) was isolated by a silica gel column and eluted by CH_2_Cl_2_/MeOH (30:1) to provide compound **13** (93.4 mg). Fr.A2 (0.6) was subjected to a silica gel column and eluted by CH_2_Cl_2_/MeOH (30:1) to give compound **12** (63.0 mg). SubFr.2 (H_2_O/EtOH, 7:3, 2.5 g) was separated by a Sephadex LH-20 column and eluted by MeOH/H_2_O (1:1) to produce five fractions (Fr.B1 to B5). Fr.B1 (0.3 g) was separated by a silica gel column and eluted by CH_2_Cl_2_/MeOH (30:1) to give compound **5** (39.4 mg). Fr.B2 was isolated by a silica gel column and eluted by CH_2_Cl_2_/MeOH (50:1) to give compound **10** (10.2 mg). Fr.B3 (0.2 g) was isolated by a silica gel column and eluted by CH_2_Cl_2_/MeOH (50:1) to give compound **9** (5.5 mg) and **11** (19.3 mg). Fr.B4 (0.4 g) was subjected to a silica gel column and eluted by CH_2_Cl_2_/MeOH (30:1) to give compounds **15** (5.1 mg) and **2** (41.5 mg). Fr.B5 (0.28 g) was separated by a silica gel column and eluted by CH_2_Cl_2_/MeOH (30:1) to provide compound **3** (9.5 mg) and **14** (41.8 mg). SubFr.3 (H_2_O/EtOH, 2:3, 2.1 g) was separated by a Sephadex LH-20 column and eluted by MeOH/H_2_O (1:1) to give two fractions (Fr.C1 to C2). Fr.C1 was isolated by a silica gel column and eluted by CH_2_Cl_2_/MeOH (50:1) to give compound **7** (692.5 mg). Fr.C2 was isolated by a silica gel column and eluted by CH_2_Cl_2_/MeOH (50:1) to give compound **4** (8.5 mg). Fr.3 (CH_2_Cl_2_/MeOH, 25:1) was further subjected to D101 macropore adsorptive resin column chromatography by successively eluted with different proportions of water/ethanol mixtures (H_2_O/EtOH, *v/v*, 7:3, 3:2, 1:19) to yield five fractions (SubFrs.5–9). SubFr.5 (H_2_O/EtOH, 3:2, 0.12 g) was subjected to silica gel column and eluted with CH_2_Cl_2_/MeOH (100:1) to give compound **16** (10.0 mg). SubFr.6 (H_2_O/EtOH, 3:2, 0.1 g) was subjected to a Sephadex LH-20 column and eluted by MeOH/H_2_O (1:1) to give compound **17** (8.9 mg). SubFr.7 (H_2_O/EtOH, 1:19, 3.0 g) was separated by silica gel column and eluted with CH_2_Cl_2_/MeOH (100:1) to give compounds **1** (26.8 mg) and compound **6** (22.5 mg). SubFr.3 (H_2_O/EtOH, 1:19, 0.12 g) was separated by a silica gel column with CH_2_Cl_2_/MeOH (100:1) as eluent to give compound **8** (2.9 mg).

*Morusone* (**1**): pale yellow amorphous powder; UV (MeOH) 270, 298.5, 332 nm; IR (KBr) ν_max_ 3385, 1655, 1617, 1434 cm^−1^; ^1^H-NMR (acetone-*d*_6_, 400 MHz) δ: 12.926 (1H, s, OH-5), 8.874 (2H, s, OH-2′, 4′), 7.217 (1H, d, *J* = 8.4 Hz, H-6′), 6.642 (1H, d, *J* = 10.0 Hz, H-1′′), 6.544 (1H, d, *J* = 2.0 Hz, H-3′), 6.486 (1H, dd, *J* = 8.4, 2.0 Hz, H-5′), 6.173 (1H, s, H-6), 6.088 (1H, s, H-4′′′), 5.812 (1H, d, *J* = 1.2 Hz, H-4′′′), 5.678 (1H, d, *J* = 10.0 Hz, H-2′′), 3.851 (2H, s, H-1′′′), 1.814 (3H, s, H-5′′′), 1.456 (6H, s, H-4′′, 5′′); ^13^C-NMR (Acetone-*d*_6_, 100 MHz) *δ*: 198.36 (C=O, C-2′′′), 182.79 (C=O, C-4), 163.42 (C, C-2), 162.69 (C, C-5), 161.97 (C, C-4′), 160.29 (C, C-7), 157.56 (C, C-2′), 153.39 (C, C-9), 145.13 (C, C-3′′′), 132.41 (CH, C-6′), 128.30 (CH, C-2′′), 125.03 (C, C-4′′′), 117.77 (C, C-3), 115.47 (CH, C-1′′), 112.23 (C, C-1′), 108.53 (CH, C-5′), 105.28 (C, C-10), 104.18 (CH, C-3′), 101.93 (C, C-8), 100.04 (CH, C-6), 78.97 (C, C-3′′), 30.50 (CH_2_, C-1′′′), 28.38 (CH_3_, C-4′′, 5′′), 17.90 (CH_3_, C-5′′′); HRESI-MS *m/z* 433.1270 [M − H]^−^ (calcd for C_25_H_21_O_7_, 433.1287).

NMR data of the known compounds **2**–**17** is provided in the Supplementary Materials.

### 3.4. Mushroom Tyrosinase Inhibitory Assay

The tyrosinase inhibitory activities of isolated compounds were determined by spectrophotometric method as described in our previous study [30]. The compounds were firstly dissolved in DMSO at a concentration of 1.0 mg/mL and then diluted to different concentrations with DMSO. Each of the sample solution (30 μL) was diluted with 970 μL of 0.05 mM sodium phosphate buffer (pH 6.8) in the test tubes, followed by the addition of 1 mL of 0.1 mg/mL l-tyrosine and finally 1.0 mL of mushroom tyrosinase solution (200 units/mL). 30 μL of DMSO and kojic acid solution were used as the blank reference and positive control, respectively. The reaction mixtures (3.0 mL) were vortexed and the initial absorbance at 492 nm was measured. After incubation for 20 min at 37 °C, the final absorbance at the same wavelength was taken. The IC_50_ values which represent the concentrations of plant extracts or compounds at which 50% of the tyrosinase activity was inhibited were determined. The percent inhibition of tyrosinase activity was calculated as follows:

(1) %Inhibition = [(*A*_2_ − *A*_1_) − (*B*_2_ − *B*_1_)]/(*A*_2_ − *A*_1_) × 100

*A*_1_ is the absorbance at 492 nm of the blank at 0 min, *A*_2_ is the absorbance at 492 nm of the blank at 20 min; *B*_1_ is the absorbance at 492 nm of test sample at 0 min, *B*_2_ is the absorbance at 492 nm of test sample at 20 min.

## 4. Conclusions

In summary, the phytochemicals in the twigs of *M. alba* were systematically studied. A total of 17 compounds, including one new compound, were isolated and their structures were determined by ESI-MS and NMR data. Among them, steppogenin, 2,4,2′,4′-tetrahydroxychalcone, morachalcone A, oxyresveratrol, and moracin M were found to exhibit significant tyrosinase inhibitory activity and were the main components responsible for the strong tyrosinase inhibitory activity, suggesting that *M. alba* twig or some of its constituents might become the promising sources in nutraceuticals and cosmeceuticals to inhibit tyrosinase activity in food products or be used in cosmetics as skin-whitening agents.

## Figures and Tables

**Table 1:** Tyrosinase inhibition activities of compounds from the twigs of *M. alba* (*n* = 3).

Compounds	IC_50_ (µM) ± SD
Morusone (**1**)	290.00 ± 7.90
Steppogenin (**2**)	0.98 ± 0.01
2,4,2′,4′-Tetrahydroxychalcone (**3**)	0.07 ± 0.02
Morachalcone A (**4**)	0.08 ± 0.02
Oxyresveratrol (**5**)	0.10 ± 0.01
Morusin (**6**)	>100
Kuwanon C (**7**)	52.00 ± 2.50
Cyclomulberrin (**8**)	66.30 ± 7.20
5,7,2′,4′-Tetrahydroxy-3-methoxyflavone (**9**)	>150
Dihydrokaempferol (**10**)	>200
Eriodictyol (**11**)	>150
2,4-Dihydroxybenzoic acid (**12**)	>200
*p*-Coumaric acid (**13**)	>200
Moracin M (**14**)	8.00 ± 0.22
Moracin J (**15**)	76.34 ± 0.86
Moracin B (**16**)	34.40 ± 1.7
Moracin D (**17**)	>200
Kojic acid	58.30 ± 1.60

## Supplementary Materials

The following are available online at: http://www.mdpi.com/1420-3049/21/9/1130/s1, the 1D (^1^H- and ^13^C-NMR), 2D NMR (^1^H-^1^H COSY, HSQC and HMBC), HRESI-MS spectra of the new compound and spectral data of known compounds.
