# Supplementary material for: Characterization of a New Flavone and Tyrosinase Inhibition Constituents from the Twigs of Morus alba L
Source: Molecules. 2016 Sep 2;21(9):1130. doi: 10.3390/molecules21091130 (PMC6274457; doi:10.3390/molecules21091130)
Supplement: Supplementary file 1 [file molecules-21-01130-s001.pdf]

Supplementary Materials: Characterization of A New Flavone and Tyrosinase Inhibition Constituents from the Twigs of *Morus alba* L.

Long Zhang, Guanjun Tao, Jie Chen and Zong-Ping Zheng

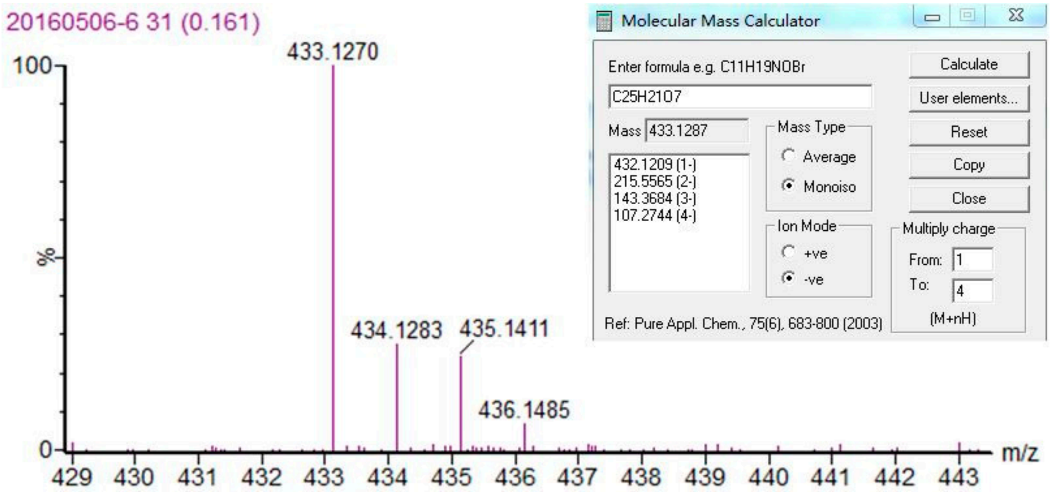

Figure S1. HR-ESI-MS data of compound **1**.

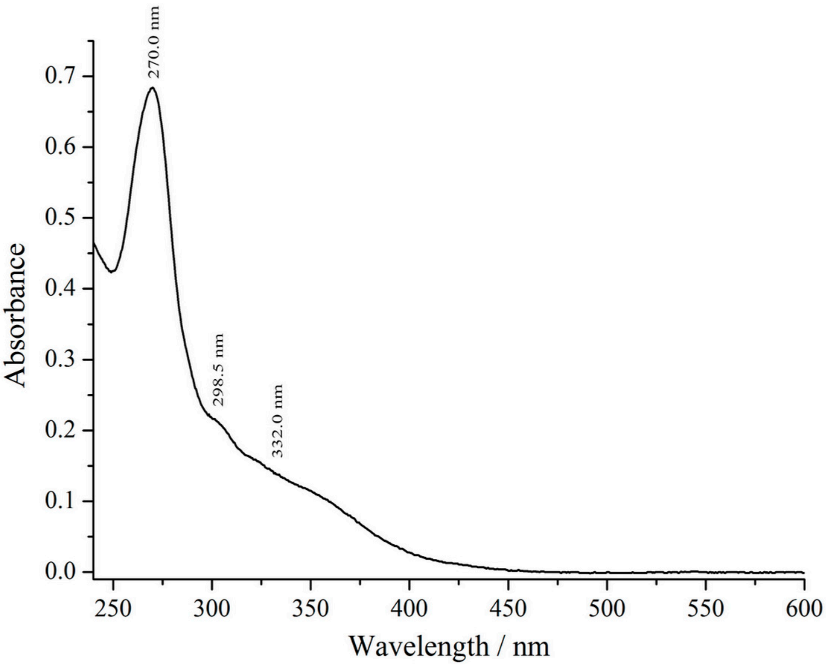

Figure S2. UV of compound **1**.

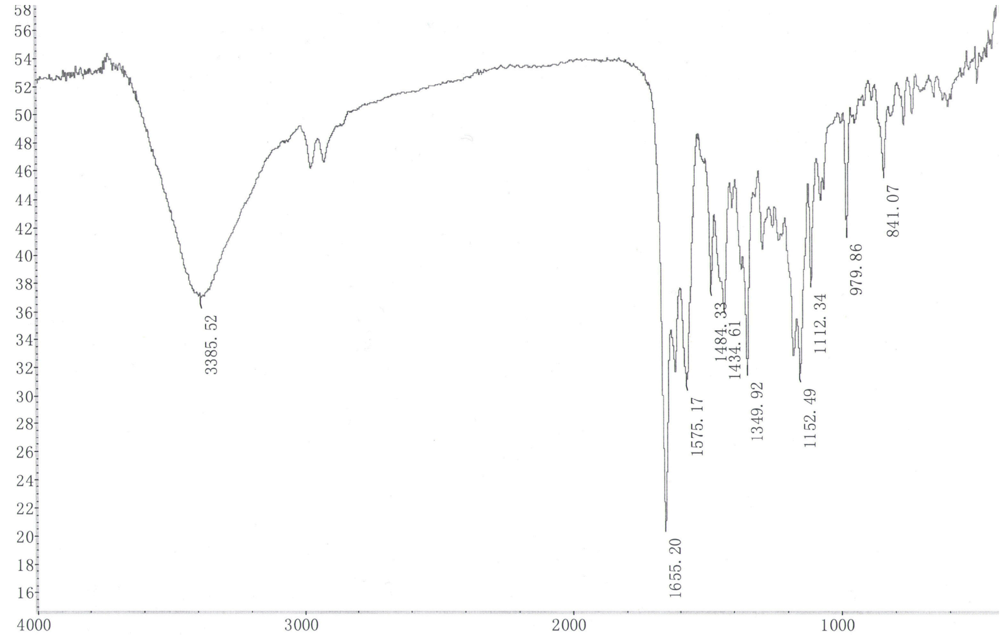

Figure S3. IR of compound **1**.

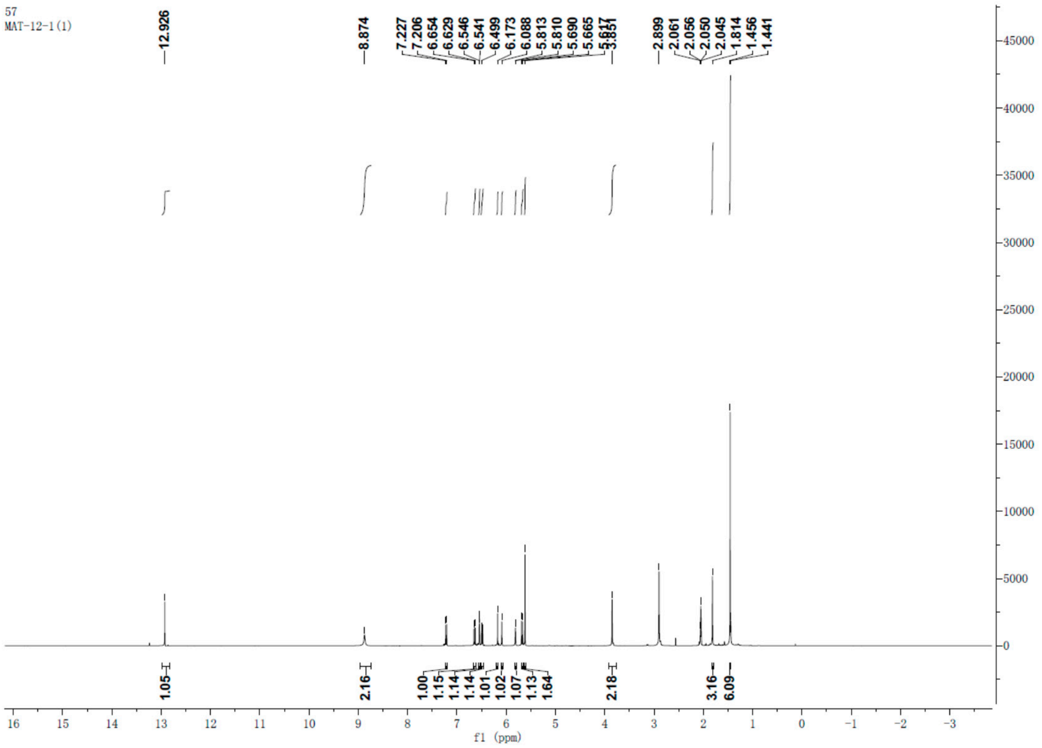

Figure S4. ^{1}H-NMR spectrum of compound **1** (in Acetone-d_6, 400 MHz).

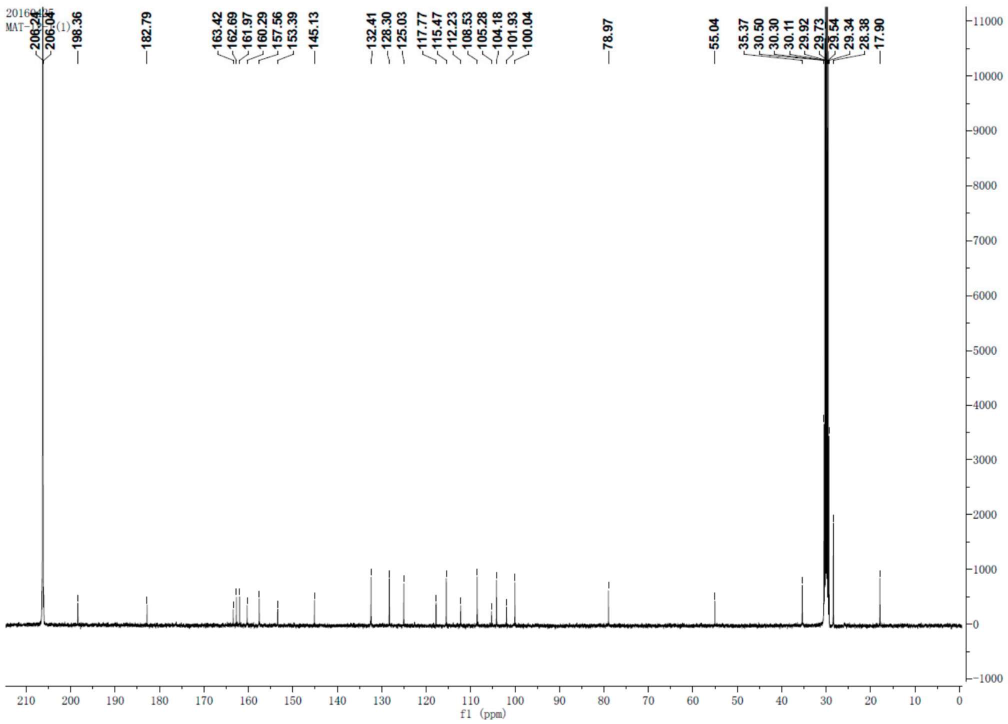

Figure S5. ^{13}C-NMR spectrum of compound **1** (in Acetone-d_6, 100 MHz).

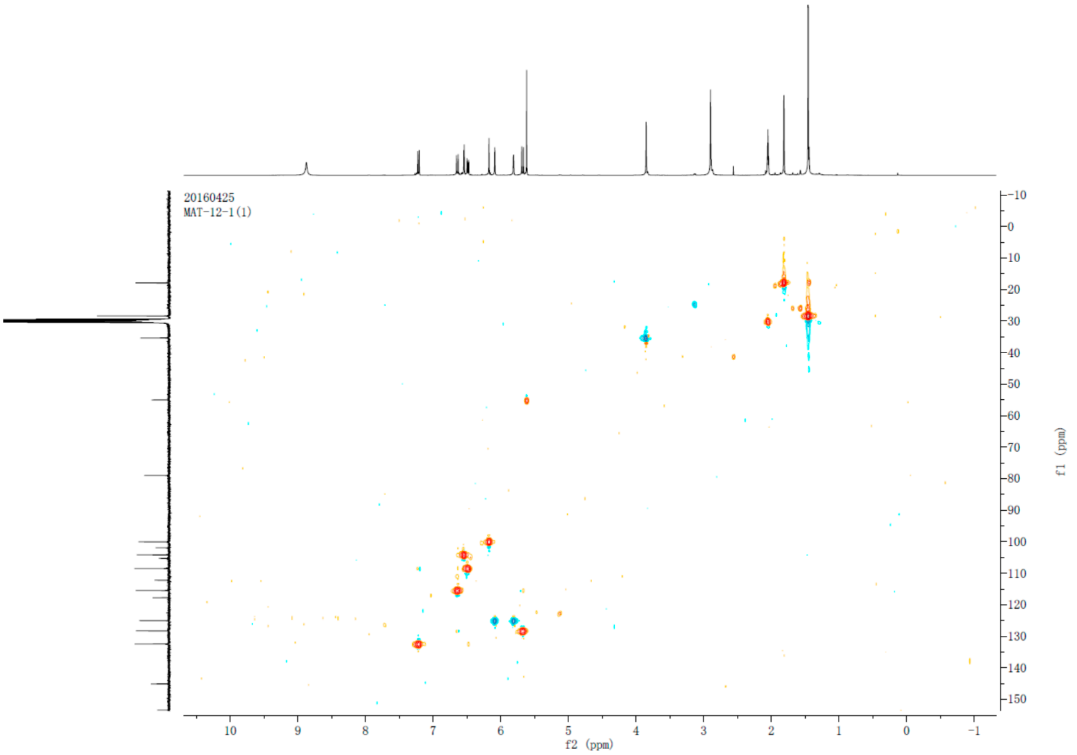

Figure S6. HSQC spectrum of compound **1** (in Acetone-d_6).

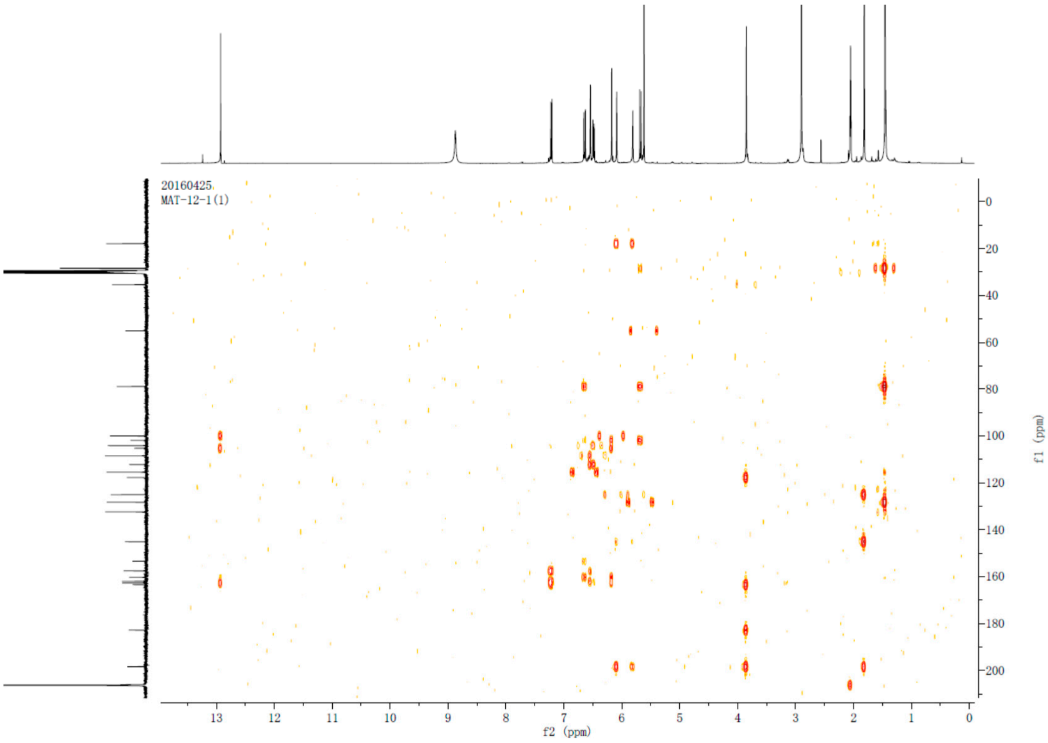

Figure S7. HMBC spectrum of compound **1** (in Acetone-*d*6).

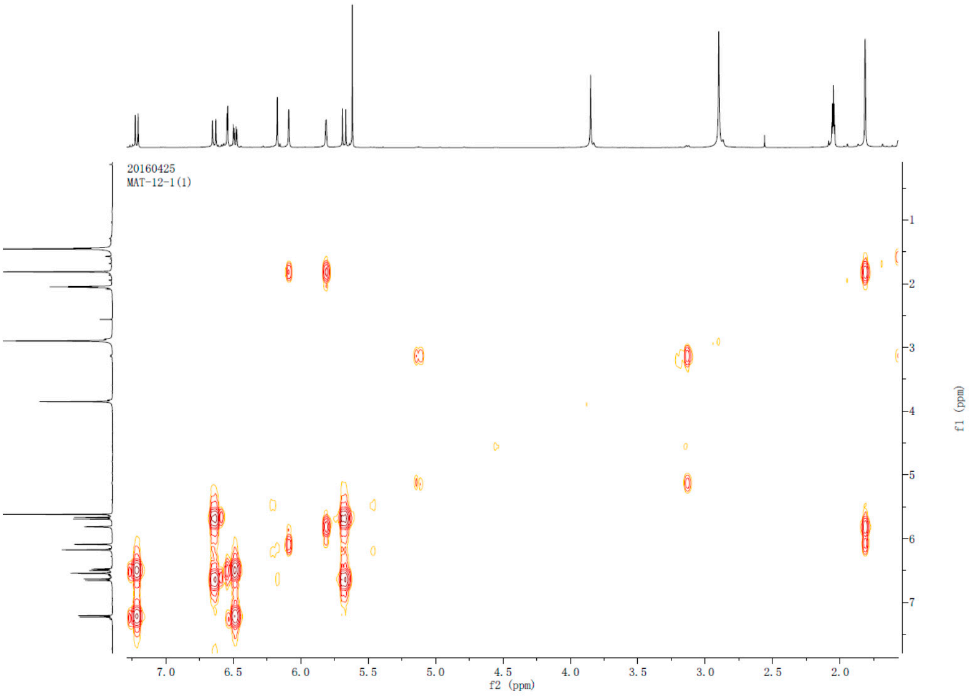

Figure S8. ^{1}H-^{1}H COSY spectrum of compound **1** (in Acetone-*d*6, 400 MHz).

NMR Data of Known Compounds

Steppogenin (**2**): ^{1}H-NMR (Acetone-d_6, 400 MHz) *δ*: 12.210 (1H, OH-5), 9.019 (2H, OH), 7.290 (1 H, d, *J* = 8.4 Hz, H-6′), 6.465 (1H, d, *J* = 2.4 Hz, H-2′), 6.415 (1H, dd, *J* = 8.4, 2.4 Hz, H-5′), 5.955 (1H, d, *J* = 2.0 Hz, H-8), 5.936 (1H, d, *J* = 2.0 Hz, H-6), 5.692 (1H, dd, *J* = 13.2, 2.8 Hz, H-2), 3.163 (1H, dd, *J* = 17.2, 13.2 Hz, H-3), 2.703 (1H, dd, *J* = 17.2, 2.8 Hz, H-3); ^{13}C-NMR (Acetone-d_6, 100 MHz) *δ*: 197.8 (C=O, C-4), 167.6 (C, C-7), 165.3 (C, C-5), 164.9 (C, C-9), 159.7 (C, C-2′), 156.5 (C, C-4′), 129.0 (CH, C-6′), 117. 3 (C, C-1′), 107.9 (CH, C-5′), 103.6 (CH, C-3′), 103.1 (C, C-10), 96.8 (CH, C-6), 95.9 (CH, C-8), 75.4 (CH, C-2), 42.7 (CH_2, C-3); ESI-MS *m*/*z* 287.1 $[M - H]^-$.

2, 4, 2′, 4′-Tetrahydroxychalcone (**3**): ^{1}H-NMR (Acetone-d_6, 400 MHz) *δ*: 13.797 (1H, s, OH-2′), 9.690 (1H, OH-4), 9.490 (1H, s, OH-2), 9.163 (1H, OH-4′), 8.211 (1H, d, *J* = 15.6 Hz, H-*α*), 8.007 (1H, d, *J* = 9.2 Hz, H-6), 7.775 (1H, d, *J* = 15.4 Hz, H-β), 7.667 (1H, d, *J* = 8.8 Hz, H-6′), 6.521 (1H, d, *J* = 2.0 Hz, H-3), 6.441 (1H, dd, *J* = 8.8, 2.4 Hz, H-5), 6.431 (1H, dd, *J* = 8.4, 2.4 Hz, H-5′), 6.345 (1H, d, *J* = 2.4 Hz, H-3′); ^{13}C-NMR (Acetone-d_6, 100 MHz) *δ*: 193.31 (C=O), 167.60 (C, C-4′), 165.62 (C, C-2′), 162.63 (C, C-4), 160.30 (C, C-2), 141.25 (CH, C-β), 132.96 (CH, C-6), 131.79 (CH, C-6′), 117.24 (CH, C-*α*), 115.17 (C, C-1′), 114.61 (C, C-1), 109.21 (CH, C-5), 108.70 (CH, C-5′), 103.86 (CH, C-3′), 103.76 (CH, C-3); ESI-MS *m*/*z* 271.1 $[M - H]^-$.

Morachalcone A (**4**): ^{1}H-NMR (Acetone-d_6, 400 MHz) *δ*: 14.155 (1H, br s, OH-2′), 9.381, 9.081 (3H, OH-4′, 2, 4), 8.213 (1H, d, *J* = 15.6 Hz, H-*α*), 7.875 (1H, d, *J* = 9.2 Hz, H-6′), 7.785 (1H, d, *J* = 15.2 Hz, H-β), 7.667 (1H, d, *J* = 8.4 Hz, H-6), 6.525 (1H, d, *J* = 2.4 Hz, H-3), 6.522 (1H, d, *J* = 8.8 Hz, H-5′), 6.444 (1H, dd, *J* = 8.4, 2.4 Hz, H-5), 5.276 (1H, m, H-2″), 3.363 (2H, d, *J* = 7.2 Hz, H-1″), 1.775, 1.638 (6H, br s, H-4″, 5″); ^{13}C-NMR (Acetone-d_6, 100 MHz) *δ*: 193.54 (C, C=O), 165.19 (C, C-4′), 162.64 (C, C-2′), 162.45 (C, C-4), 160.13 (C, C-2), 140.97 (CH, C-β), 131.79 (CH, C-6′), 131.44 (C, C-3″), 129.98 (CH, C-6), 123.53 (CH, C-2″), 117.56 (CH, C-*α*), 116.15 (C, C-1), 115.29 (C, C-3′), 114.56 (C, C-1′), 109.23 (CH, C-5), 108.00 (CH, C-5′), 103.76 (CH, C-3), 25.96 (CH2, C-1″), 22.40 (CH_3, C-4″), 18.01 (CH_3, C-5″); ESI-MS *m*/*z* 339.1 $[M - H]^-$.

Oxyresveratrol (**5**): ^{1}H-NMR (400 MHz, CD_3OD) *δ*: 7.32 (1H, d, *J* = 9.1 Hz, H-6), 7.26 (1H, d, *J* = 16.4 Hz, H-7), 6.81 (1H, d, *J* = 16.4 Hz, H-8), 6.45 (2H, d, *J* = 2.0 Hz, H-2′, 6′), 6.31 (2H, overlap, H-3, 5), 6.14 (1H, t, *J* = 2.0 Hz, H-4′). ^{13}C-NMR (100 MHz, CD_3OD) δ: 159.8 (C, C-3′, 5′), 159.4 (C, C-4), 157.6 (C, C-2), 142.5 (C, C-1′), 128.7 (CH, C-6), 126.8 (CH, C-8), 125.1 (CH, C-7), 118.1 (C, C-1), 108.7 (CH, C-5), 106.0 (CH, C-2′, 6′), 103.9 (CH, C-3), 102.6 (CH, C-4′); ESI-MS *m*/*z* 243.1 $[M - H]^-$.

Morusin (**6**): ^{1}H-NMR (Acetone-d_6, 400 MHz) *δ*: 13.233 (1H, s, OH-5), 8.861 (2H, s, OH-2′, 4′), 7.250 (1H, d, *J* = 8.4 Hz, H-6′), 6.589 (1H, d, *J* = 9.6 Hz, H-1″), 6.575 (1H, overlapped, H-3′), 6.529 (1H, dd, *J* = 8.4, 2.4 Hz, H-5′), 6.148 (1H, s, H-6), 5.616 (1H, d, *J* = 10.0 Hz, H-1″), 5.129 (1H, m, H-2‴), 3.134 (2H, d, *J* = 6.8 Hz, H-1‴), 1.567, 1.436 (6H, s, H-4‴, 5‴), 1.425 (6H, s, H-4″, 5″); ^{13}C-NMR (Acetone-d_6, 100 MHz) *δ*: 183.27 (C=O, C-4), 162.77 (C, C-7), 162.44 (C, C-5), 161.62 (C, C-2′), 160.0 (C, C-2), 157.41 (C, C-4′), 153.29 (C, C-9), 132.47 (CH, C-6′), 132.34 (C, C-3‴), 127.99 (CH, C-2″), 122.56 (CH, C-2‴), 121.79 (C, C-3), 115.47 (CH, C-1″), 112.78 (C, C-1′), 108.26 (CH, C-5′), 105.64 (C, C-10), 104.02 (CH, C-3′), 101.66 (C, C-8), 99.81 (CH, C-6), 78.78 (C, C-3″), 28.34 (CH_3, C-4‴, 5‴), 25.88, 17.75 (CH_3, C-4″, 5″), 24.69 (CH_2, C-1‴); ESI-MS *m*/*z* 419.2 $[M - H]^-$.

Kuwanon C (**7**): ^{1}H-NMR (Acetone-d_6, 400 MHz) *δ*: 13.085 (1H, s, OH-5), 9.005 (3H, br s, OH-7, 2′, 4′), 7.219 (1H, d, *J* = 8.4 Hz, H-6′), 6.583 (1H, d, *J* = 2.0 Hz, H-3′), 6.526 (1H, dd, *J* = 8.4, 2.0 Hz, H-5′), 6.331 (1H, s, H-6), 5.206, 5.137 (1H, m, H-2″, 2‴), 3.35 (2H, d, *J* = 7.3 Hz, H-1″ or 1‴), 3.362 (1H, d, *J* = 6.4 Hz, H-1″ or 1‴), 3.135 (1H, d, *J* = 6.0 Hz, H-1″ or 1‴), 1.581, 1.578, 1.558, 1.428 (12H, s, H-4″, 4‴, 5″, 5‴); ^{13}C-NMR (Acetone-d_6, 100 MHz) *δ*: 183.37 (C=O, C-4), 162.39 (C, C-7), 161.82 (C, C-5), 161.37 (C, C-2′), 160.80 (C, C-2), 157.30 (C, C-4′), 156.52 (C, C-9), 132.33 (CH, C-6′), 132.07, 131.63 (C, C-3″, 3‴), 123.17, 122.77 (CH, C-2″, 2‴),

121.18 (C, C-3), 113.12 (C, C-1′), 108.07 (CH, C-5′), 106.78 (C, C-8), 105.23 (C, C-10), 103.90 (CH, C-3′), 98.84 (CH, C-6), 25.92, 25.87 (CH_3, C-4″, 4‴), 24.62 (CH_2, C-1″), 22.11 (CH_2, C-1‴), 17.79, 17.72 (CH_3, C-5″, 5‴); ESI-MS *m*/*z* 421.1 $[M - H]^-$.

Cyclomulberrin (**8**): ^{1}H-NMR (Acetone-*d*$_6$, 400 MHz) *δ*: 12.804 (1H, s, OH-5), 9.664 (1H, s, OH-7), 9.430 (1H, s, OH-4′), 7.715 (1H, d, *J* = 8.8 Hz, H-6′), 6.652 (1H, dd, *J* = 8.4, 2.4 Hz, H-5′), 6.435 (1H, d, *J* = 2.0 Hz, H-3′), 6.326 (1H, s, H-6), 6.191 (1H, d, *J* = 9.6 Hz, H-1″), 5.470 (1H, m, H-2″), 5.308 (1H, m, H-2‴), 3.523 (2H, m, H-1‴), 1.933, 1.676 (6H, s, H-4″, 5″), 1.831, 1.656 (6H, s, H-4‴, 5‴); ^{13}C-NMR (Acetone-*d*$_6$, 100 MHz) *δ*: 179.52 (C=O, C-4), 164.08 (C, C-4′), 162.09 (C, C-7), 161.08 (C, C-5), 159.16 (C, C-2′), 156.49 (C, C-2), 155.35 (C, C-9), 138.9 (C, C-3″), 132.09 (C, C-3‴), 126.30 (CH, C-6′), 123.52 (CH, C-2″), 122.26 (CH, C-2″), 111.92 (CH, C-5′), 109.79 (C, C-1′), 108.79 (C, C-3), 107.62 (C, C-8), 105.61 (C, C-10), 105.02 (CH, C-3′), 99.47 (CH, C-6), 70.47 (CH, C-1″), 25.94 (CH_3, C-4″, 5″), 22.33 (CH_2, C-1‴), 18.73, 18.21 (CH_3, C-4″, 5″); ESI-MS *m*/*z* 419.1 $[M - H]^-$.

5,7,2′,4′-Tetrahydroxy-3-methoxyflavone (**9**): ^{1}H-NMR (Acetone-*d*$_6$, 400 MHz) *δ*: 12.759 (1H, s, OH-5), 8.970 (2H, OH), 7.401 (1H, d, *J* = 9.2 Hz, H-6′), 6.552 (1H, dd, *J* = 9.2, 2.4 Hz, H-5′), 6.543 (1H, d, *J* = 2.4 Hz, H-3′), 6.402 (1H, d, *J* = 2.0 Hz, H-8), 6.262 (1H, d, *J* = 2.0 Hz, H-6); ^{13}C-NMR (Acetone-*d*$_6$, 100 MHz) *δ*: 179.30 (C=O, C-4), 164.97 (C, C-7), 163.40 (C, C-5), 162.21 (C, C-4′), 158.59 (C, C-9), 157.82 (C, C-2), 139.61 (C, C-3), 132.55 (CH, C-6′), 110.80 (C, C-1′), 108.73 (CH, C-5′), 106.26 (C, C-10), 104.58 (CH, C-3′), 99.45 (CH, C-6), 94.65 (CH, C-8), 61.09 (CH_3, OCH_3-3); ESI-MS *m*/*z* 315.1 $[M - H]^-$.

Dihydrokaempferol (**10**): ^{1}H-NMR (Acetone-*d*$_6$, 400 MHz) *δ*: 11.701 (1H, s, OH-5), 9.699 (1H, s, OH-7), 8.541 (1H, s, OH-4′), 7.421 (2H, d, *J* = 8.4 Hz, H-2′, 6′), 6.896 (2H, d, *J* = 8.4 Hz, H-3′, 5′), 5.996 (1H, d, *J* =2.0 Hz, H-8), 5.951 (1H, d, *J* = 2.0 Hz, H-6), 5.304 (1H, d, *J* = 11.6 Hz, H-2), 4.680 (1H, d, *J* = 4.2 Hz, OH-3), 4.659 (1H, dd, *J* = 11.6, 4.2 Hz, H-3); ^{13}C-NMR (Acetone-*d*$_6$, 100 MHz) *δ*: 198.37 (C=O), 167.89 (C, C-7), 165.08 (C, C-9), 164.08 (C, C-5), 158.92 (C, C-4′), 130.39 (CH, C-2′, 6′), 129.21 (C, C-1′), 116.00 (CH, C-3′, 5′), 101.63 (C, C-10), 97.18 (CH, C-6), 96.16 (CH, C-8), 84.44 (CH, C-2), 73.21 (CH, C-3); ESI-MS *m*/*z* 287.0 $[M - H]^-$.

Eriodictyol (**11**): ^{1}H-NMR (Acetone-*d*$_6$, 400 MHz) *δ*: 12.169 (1H, OH-5), 8.553 (2H, s, OH-3′,4′), 7.033 (1 H, s, H-2′), 6.868 (1H, overlapped, H-5′, 6′), 5.958 (1H, d, *J* = 2.0 Hz, H-8), 5.943 (1H, d, *J* = 2.0 Hz, H-6), 5.390 (1H, dd, *J* = 12.8, 3.2 Hz, H-2), 3.133 (1H, dd, *J* = 17.2, 12.8 Hz, H-3), 2.725 (1H, dd, *J* = 17.2, 3.2 Hz, H-3); ^{13}C-NMR (Acetone-*d*$_6$, 100 MHz) *δ*: 197.3 (C=O, C-4), 166.6 (s, C-7), 167.4 (C, C-5), 165.3 (C, C-9), 164.4 (C, C-5), 146.5 (C, C-4′), 146.1 (C, C-3′), 131.7 (C, C-1′), 119. 3 (CH, C-6′), 116.1 (CH, C-2′), 114.8 (CH, C-5′), 103.3 (C, C-10), 96.9 (CH, C-6), 95.9 (CH, C-8), 80.0 (CH, C-2), 43.6 (CH_2, C-3); ESI-MS *m*/*z* 287.0 $[M - H]^-$.

2,4-Dihydroxybenzoic acid (**12**): ^{1}H-NMR (Acetone-*d*$_6$, 400 MHz) *δ*: 7.704 (1H, d, *J* = 8.8 Hz, H-6), 6.403 (1H, dd, *J* = 8.8, 2.4 Hz, H-5), 6.350 (1H, d, *J* =2.4 Hz, H-3); ^{13}C-NMR (Acetone-*d*$_6$, 100 MHz) *δ*: 173.13 (C=O), 165.26 (C, C-4), 165.19 (C, C-2), 132.90 (CH, C-6), 108.73 (CH, C-5), 105.50 (C, C-1), 103.34 (CH, C-3); ESI-MS *m*/*z* 153.0 $[M - H]^-$.

p-Coumaric acid (**13**): ^{1}H-NMR (Acetone-*d*$_6$, 400 MHz) *δ*: 8.879 (1H, s, OH-4), 7.626 (1H, d, *J* = 16.0 Hz, H-8), 7.549 (2H, d, *J* = 8.4 Hz, H-2, 6), 6.898 (2H, d, *J* = 8.8 Hz, H-3, 5), 6.344 (1H, d, *J* = 16.0 Hz, H-7); ^{13}C-NMR (Acetone-*d*$_6$, 100 MHz) *δ*: 168.63 (C=O, C-9), 160.60 (C, C-4), 145.84 (CH, C-7), 131.01 (CH, C-2, 6), 127.14 (C, C-1), 116.78 (CH, C-3, 5), 115.75 (CH, C-8); ESI-MS *m*/*z* 163.0 $[M - H]^-$.

Moracin M (**14**): ^{1}H-NMR (Acetone-*d*$_6$, 400 MHz) *δ*: 8.60 (3H, br s, OH), 7.40 (1H, d, *J* = 8.4 Hz, H-4), 7.20 (1H, s, H-3), 6.99 (1H, d, *J* = 2.0 Hz, H-7), 6.86 (2H, d, *J* = 2.0 Hz, H-2′, 6′), 6.81(1H, dd, *J* = 8.4, 2.0 Hz, H-5), 6.37 (1H, t, *J* = 2.0 Hz, H-4′); ^{13}C-NMR (Acetone-*d*$_6$, 100 MHz) *δ*: 159.83 (C, C-3′, 5′), 156.78 (C, C-7a), 156.71 (C, C-2), 155.58 (C, C-6), 133.47 (C, C-1′), 100.8 (CH, C-3), 122.65 (C, C-3a), 122.09 (CH, C-4), 113.31 (CH, C-5), 98.51 (CH, C-3), 103.92 (CH, C-2′, 6′), 103.61 (CH, C-7), 102.42 (CH, C-4′); ESI-MS *m*/*z* 241.0 $[M - H]^-$.

Moracin J (**15**): ^{1}H-NMR (Acetone-d_6, 400 MHz) δ: 8.459 (1H, OH), 7.177 (1H, s, H-4), 7.005 (1H, s, H-7), 6.982 (1H, d, J = 0.8 HZ, H-3), 6.852 (2H, d, J = 2.4 Hz, H-2′, 6′), 6.365 (1H, t, J = 2.4 Hz, H-4′), 3.927 (3H, s, OCH_3); ^{13}C-NMR (Acetone-d_6, 100 MHz) δ: 159.9 (C, C-3′, 5′), 156.0 (C, C-2), 150.0 (C, C-7a), 147.6 (C, C-5), 144.9 (C, C-6), 133.6 (C, C-1′), 122.7 (C, C-3a), 105.8 (CH, C-4), 103.8 (CH, C-2′, 6′), 103.6 (CH, C-4′), 102.5 (CH, C-3), 95.9 (CH, C-7), 56.8 (CH_3, OCH_3); ESI-MS *m*/*z* 271.1 $[M - H]^-$.

Moracin B (**16**): ^{1}H-NMR (Acetone-d_6, 400 MHz) δ: 7.192 (1H, s, H-4), 7.061 (1H, d, J = 0.4 HZ, H-3), 7.011 (1H, s, H-7), 6.946 (1H, dd, J = 2.4, 2.0 Hz, H-2′), 6.921 (1H, ds, J =2.4, 2.0 Hz, H-6′), 6.409 (1H, t, J = 2.2 Hz, H-4′), 3.934 (3H, s, OCH_3), 3.826 (3H, s, OCH_3); ^{13}C-NMR (Acetone-d_6, 100 MHz) δ: 162.42 (C, C-3′), 159.9 (C, C-5′), 155.77 (C, C-2), 150.2 (C, C-7a), 147.71 (C, C-5), 144.91 (C, C-6), 133.60 (C, C-1′), 122.66 (C, C-3a), 105.85 (CH, C-4), 104.82 (CH, C-2′) 102.81 (CH, C-6′), 102.27 (CH, C-4′), 102.13 (CH, C-3), 95.90 (CH, C-7), 56.78 (CH_3, OCH_3), 55.70 (CH_3, OCH_3); ESI-MS *m*/*z* 285.1 $[M - H]^-$.

Moracin D (**17**): ^{1}H-NMR (Acetone-d_6, 400 MHz) δ : 8.839 (1H, s, OH-6), 8.740 (1H, s, OH-5′), 7.451 (1H, d, J = 8.4 Hz, H-4), 7.005 (1H, d, J = 2.0 Hz, H-7), 6.867 (1H, d, J = 0.8 Hz, H-3), 6.837 (1H, dd, J = 8.4, 2.0 Hz, H-5), 6.831 (1H, d, J = 10.0 Hz, H-1″), 6.793 (1H, d, J = 2.4 Hz, H-6′), 6.344 (1H, d, J = 2.4 Hz, H-2′), 5.668 (1H, d, J = 10.0 Hz, H-2″), 1.412 (6H, s, H-4″, 5″); ^{13}C-NMR (Acetone-d_6, 100 MHz) δ: 159.17 (C, C-3′), 157.03 (C, C-5′), 156.87 (C, C-2), 156.22 (C, C-7a), 154.25 (C, C-6), 129.50 (C, C-1′), 128.90 (CH, C-2″), 122.34 (C, C-4′), 122.20 (CH, C-2′), 122.4 (C, C-1), 121.29 (CH, C-4), 113.43 (CH, C-1″), 112.19 (C, C-3a), 108.16 (CH, C-5), 106.69 (CH, C-6′), 104.83 (CH, C-3), 98.0 (CH, C-7), 77.9 (C, C-3″), 27.8 (CH3, C-4″, 5″); ESI-MS *m*/*z* 307.1 $[M - H]^-$.
